# Expanding Rehabilitation Options for Dysphagia: Skill-Based Swallowing Training

**DOI:** 10.1007/s00455-022-10516-3

**Published:** 2022-09-12

**Authors:** Maggie-Lee Huckabee, Ruth Flynn, Madeline Mills

**Affiliations:** The Rose Centre for Stroke Recovery and Research, School of Psychology Speech and Hearing, The University of Canterbury, Leinster Chambers, Level One, 249 Papanui Road, Strowan, Christchurch, 8052 New Zealand

**Keywords:** Dysphagia, Deglutition, Motor control, Cortex, Skill, Learning

## Abstract

Over the past four decades, our understanding of swallowing neural control has expanded dramatically. However, until recently, advances in rehabilitation approaches for dysphagia have not kept pace, with a persistent focussing on strengthening peripheral muscle. This approach is no doubt very appropriate for some if not many of our patients. But what if the dysphagia is not due to muscles weakness? The purpose of this clinical manuscript is to reflect on where we have been, where we are now and perhaps where we need to go in terms of our understanding of swallowing motor control and rehabilitation of motor control impairments. This compilation is presented to clinicians in the hope that suggesting approaches “outside the box” will inspire clinicians to focus their attention “inside the box” to ultimately improve rehabilitation and long-term outcomes for patients with dysphagia.

## Introduction

Dysphagia is a prevalent condition that is often associated with both congenital and acquired neurological impairment and structural disorders effecting oropharyngeal function [1–3]. Swallowing difficulties can range from total inability to elicit a pharyngeal response or airway protection to slight discomfort when consuming food and liquid. Resulting adverse effects include dehydration, malnutrition, impact on quality of life, and pneumonia which can significantly increase risk of mortality [4–9].

Historically, dysphagia management has focused heavily on compensatory strategies to improve symptoms, or muscle strengthening exercises. One might predict, therefore, that the majority of dysphagic patients must present with significant weakness, particularly since swallowing does not require maximal muscle strength [10, 11]. Research suggests, however, that weakness is not always the underlying cause of dysphagia [12, 13] which validates further inquiry into the efficacy of these approaches for the majority of dysphagic patients. Additionally, only a minority of the recommended strengthening exercises have adequate evidence for long-term improvement in swallowing [14] and, indeed, some evidence suggests that strength training poses potential adverse effects [15]. Fortunately, over the past decade, research has focused on the imperative role of cortical control of swallowing [16–18]. This research is opening doors to exploration of different approaches to rehabilitation that concentrate on modulating cortical activity to improve deglutition.

The purpose of this manuscript is to reflect on where we have been, where we are now and perhaps where we need to go in terms of our understanding of swallowing motor control and rehabilitation of motor control impairments. This compilation is presented in the hope that suggesting approaches “outside the box” will inspire clinicians to focus their attention “inside the box” to ultimately improve rehabilitation and long-term outcomes for patients with dysphagia.

## An Historical Prospective

The foundations of our current understanding of swallowing neural control were proposed several decades ago and were based largely on fictive, electrically stimulated swallowing in experimental animal studies [19–22]. Such research was instrumental in defining basic constructs of neural input underlying deglutitive behavior and provided the foundations for early models of swallowing motor control. This research identified and elaborated on a brainstem-driven central pattern generator (CPG), consisting of the nucleus tractus solitarius (NTS), the adjacent reticular formation, and the nucleus ambiguus (NA) [19–22]. This construct presented pharyngeal swallowing as a largely, if not exclusively, reflexive motor task with involvement of cortical control limited to initiation of the pharyngeal reflex [19]. This thinking was evident in the models of swallowing neural control proposed at the time, such as that by Jean [20] which detailed a swallowing network isolated to the medullary structures and with no cortical influence.

The contribution of this research cannot be understated, and has provided a solid foundation for exploring swallowing motor control over the ensuing four decades. Clinical management of dysphagia in those early years reflected our understanding of swallowing neural control: pharyngeal swallowing was considered a brainstem-driven reflex, and was therefore not amenable to change. Consistent with this presumption, approaches to dysphagia management at that time were characterised by compensatory techniques. Postural changes such as the chin tuck posture, as described by Logemann [23], or the head turn manoeuvre, as described by Kirchner [24], were utilised to manipulate physical dimensions to redirect bolus flow and structurally maximise swallowing efficiency in the presence of an ‘impaired reflex’. Other compensatory measures included (and, to this day, still include) manipulation of the bolus itself, such as in modification of dietary textures via thickening of liquids or pureeing of solids, under the same premise that compensating for impairment might be the best, if not only, available management option. Compensatory approaches continue to be frequently and appropriately applied in management of dysphagia.

Direct rehabilitation strategies—such as peripheral muscle strengthening therapies—gained popularity in the 90s [25–30]. Such approaches were enveloped within the perspective that although the swallowing reflex was viewed as not amenable to change, perhaps the muscular substrates responsible for its execution could be. As such, several peripheral muscle strengthening exercises emerged into dysphagia rehabilitation. Initially described as compensatory measures, approaches such as the Mendelsohn manoeuvre and effortful swallow were carried over to the muscle strengthening domain [28, 31]. Additional muscle strengthening exercises were also developed and integrated into practice, including the head-lift manoeuvre [32], expiratory muscle strength training (EMST) [33], and the tongue-hold manoeuvre [34]. With few exceptions, dysphagia rehabilitation strategies coming into this century were focused on muscle strengthening, limited by the perception of swallowing as a reflexive motor behaviour.

This thinking was challenged by Martin and Sessle [35] who reviewed clinical, neuroanatomical, and neurophysiological studies to propose the significant role of the cerebral cortex in swallowing. They noted that swallowing impairment following cortical dysfunction was a well-recognised clinical phenomenon and discussed several studies that supported this connection, including lesion studies in which ablation of various cortical areas resulted in various dysphagic symptoms. The authors went on to explore the literature regarding cortical stimulation and its subsequent evocation and modulation of swallowing behaviours. With this paper came the beginning of a significant shift in understanding: ingestive swallowing is not possible with the brainstem alone. It requires a cortex.

With direction from Martin and Sessle [35] researchers increased consideration of alternate models of swallowing neural control with an emphasis on cortical modulation of the brainstem-driven CPG. Jean [36] proposed an updated model of swallowing neurology in which they maintained the significant importance of the CPG within the medulla, although they also incorporated and discussed the role of supramedullary structures in execution and modulation of swallowing. Ertekin [16] further contributed, with a model that integrated the additional decade of neuroimaging research since the early publication of Jean [36]. As this research advanced, so did the complexity of the models representing cortical input in swallowing. Additional cortical and supramedullary input in CPG modulation was proposed, such as that from the extrapyramidal, limbic, and cerebellar systems. Eventually, Daniels et al. [37] presented a model that framed swallowing with predominant cortical generation, discussing several cortical and subcortical areas that descend to the brainstem and modulate the pharyngeal response. More recently, the role of white matter structures—specifically, the pyramidal tract, corona radiata, internal capsule, corpus callosum, and superior longitudinal fasciculus—have also been identified as playing crucial roles in the neural control of swallowing [38, 39].

What remains unclear, however, is whether this role of the cortex is to *modulate* the pharyngeal CPG for ingestive swallowing, or whether there exists a *unique* swallowing neural network for ingestive swallowing that is differentiated from the reflexive CPG. Studies have investigated neural activity during both voluntary and reflexive swallowing and revealed that, compared to reflexive swallowing, voluntary swallowing is associated with activation of a greater number of cortical regions [40, 41]. While separate models differentiating the swallowing neural networks between these conditions are yet to be developed, these data suggest that ingestive swallowing, which by nature is at a minimum volitionally executed, may be controlled quite differently than naïve reflexive swallowing behaviour. Future research is indicated in this area to advance this understanding. This point is crucial as greater cortical activation during prandial swallowing allows greater options for rehabilitation.

Coinciding with the growing recognition of cortical contribution to swallowing was the growing understanding of mechanisms of neuroplasticity and how such principles might apply to dysphagia management. Kleim and Jones [42] defined neuroplasticity as “the mechanism by which the brain encodes experiences and learns new behaviours” as well as that by which it “relearns lost behaviours in response to rehabilitation” after damage (p. S225). They proposed ten key principles of experience-dependent neuroplasticity in the context of neuroscientific literature and application to clinical rehabilitation. As such, these principles are thought to be central in understanding and engagement of effective rehabilitation for those with neurological injury. Robbins et al. [43] translated these principles to dysphagia management practices and emphasised their role in addressing critical issues in dysphagia recovery and rehabilitation. Publications by Kleim and Jones [42] and Robbins and colleagues [43] mark a further theoretical weakening in the perception of ingestive behaviour as a reflex that is not amenable to rehabilitation.

## Expanding Options for Rehabilitation of Dysphagia

However, despite the now widely accepted understanding of cortical contribution to swallowing and concurrent literature detailing capacity for neuroplastic change, a largely outdated approach to dysphagia management predominates. Until recently, a persisting feature in published intervention approaches is the aim of increasing muscle strength: consider exercises such as the tongue-hold manoeuvre [34], effortful swallow [28], Mendelsohn manoeuvre [31], head-lift manoeuvre [32], or EMST [33]. Weakness is an inarguable component of dysphagia secondary to several aetiologies. In cases of lower motor neuron damage or sarcopenia, and in potentially many cases of upper motor neuron impairment, strengthening exercises are generally sensible approaches for rehabilitation of such weakness. However, one must ask the question: is weakness *always* the underlying cause of dysphagia? Given the degree of cortical modulation now evident in swallowing neural control, it might be assumed that the answer to this question is negative. While post-swallow pharyngeal residual evident on videofluoroscopic imaging may be secondary to weakness of the pharyngeal musculature, it may also be secondary to impaired motor planning or execution of muscle activation due to deficits at the cortical level. The concept of swallowing apraxia is not novel and has been described as the inability to initiate or organise the swallowing motor sequence despite adequate range of motion in the relevant musculature [44]. This is a tricky description given that the longstanding definition of apraxia relates specifically to impairment in execution of skilled motor task not associated with other impairments. Therefore use of this definition supports the evolving understanding that swallowing is a skilled, purposeful movement and therefore might be susceptible to apraxic impairment [45]. In a single-patient case study of an individual with swallowing apraxia secondary to recurrent ischemic strokes, Yun et al. [46] emphasised the need for clinicians to consider apraxia in differential diagnosis. However, further research is undoubtedly needed to define conditions such as apraxia, ataxia and spasticity as they may apply to swallowing before application leads to misuse. Regardless, in the case where muscle weakness is not the cause of impaired biomechanics, rehabilitation approaches are required that shift the focus away from the muscle and into the brain. This has led to a recent exploration of approaches that are designed to modulate or adapt the pharyngeal swallowing motor plan through increased cortical activation.

## Non-invasive Brain Stimulation for Swallowing Recovery

Non-invasive brain stimulation (NIBS) has been applied to swallowing rehabilitation to stimulate the cortex and promote improved outcomes. Growing research focuses on the use of transcranial magnetic stimulation (TMS) and transcranial direct current stimulation (tDCS), to modulate neuroplasticity in the treatment of dysphagia [47]. When applied centrally (to the cortex or cerebellum) these neurostimulation techniques are thought to increase synaptic efficiency by establishing new and/or reinforcing impaired neural connections for functional recovery following injury [48].

TMS provides safe, controlled activation of the cortex using electromagnetic induction to underlying neural structures the brain [49]. When TMS is provided repetitively (repetitive TMS or rTMS) it can increase or decrease cortical excitability beyond the duration of stimulation [49]. In contrast, tDCS delivers direct currents through electrodes on the scalp to modify transmembrane potentials and change cortical excitability [49]. Evidence suggests that these techniques can be used to induce long-lasting plasticity changes in the pharyngeal motor cortex which may modulate swallowing behaviour [50]. Indeed, some systematic reviews and meta-analyses have reported significant effect size when evaluating the efficacy of NIBS to improve impaired swallowing [51–53].

Cheng et al. [51] conducted a systematic search to investigate the effects of rTMS and tDCS on swallowing related outcomes in post-stroke dysphagia. Data from 852 patients was synthesised from twenty-six RCTs, comparing neurostimulation with placebo stimulation or standard care [51]. The primary outcome measure was change in any relevant clinical swallowing behaviour [51]. The respective studies utilised a range of outcome measures including: the Penetration Aspiration Scale (PAS) [54], Dysphagia Outcome and Severity Scale (DOSS) [55], Mann Assessment of Swallowing Ability (MASA) [56], Functional Dysphagia Scale (FDS) [57]. Meta-analyses found that active neurostimulation treatments showed a significant and moderate effect size compared to control treatments (0.69 [95% CI = 0.50, 0.89]; *p* < 0.001) with treatment effects strongest in acute (< 14 days) stroke patients (0.8 [95% CI = 0.34, 1.26]; *p* < 0.001) [51]. Both rTMS and tDCS showed similar effect sizes within the first 2 weeks, although no significant treatment effects were reported beyond three months [51]. In terms of effects based on stimulation hemisphere, bihemispheric stimulation appeared to be most effective (0.93 [95% CI = 0.53, 1.33]; *p* < 0.001) [51]. Yet, the most beneficial hemisphere for unilateral stimulation differed between methods. Specifically, unilateral rTMS using ipsilesional high-frequency stimulation had a combined effect size of 0.83 (95% CI = 0.14, 1.52; *p* = 0.02). Whereas, for tDCS a significant effect size was found only with anodal stimulation applied over the contralesional hemisphere (1.04 [95% CI = 0.54, 1.53]; *p* < 0.001) [51]. Overall, these findings provide a platform for future research and clinical practice while demonstrating that rTMS and tDCS have positive effects for post-stroke dysphagic patients compared to traditional management or sham stimulation [51]. However, due to the patient heterogeneity, the authors posit that future studies should investigate neurostimulation protocols tailored to patients’ individual characteristics and prognosis [51].

Cheng & Hamdy [49] further summarise findings on the effects of rTMS and tDCS to induce neuroplasticity in dysphagia rehabilitation while discussing the variability in patient responsiveness to NIBS. While considering the preliminary positive findings of neurostimulation to improve dysphagia, this review highlights some of the limitations [49]. For example, factors such as genetic variability and/or brain configuration across patients, as well as differences in the level of brain activation prior to stimulation may impact the treatment outcomes [49].

The foundational work of Kleim and Jones [42] identifies inherent shortcomings associated with neurostimulation as a treatment approach for dysphagia. Their paper reports on the fundamental principles of experience-dependent neural plasticity to reorganise the brain and restore lost function [42]. In essence, the authors propose that rehabilitation must incorporate learning experiences to promote adaptive neural changes following injury [42]. Quite clearly, NIBS takes a very different approach by stimulation of cortical tissue in a cortical area considered to be specific to swallowing in general. However, this approach is not specific to identified pathophysiology that characterises the impairment. It is a more ‘one size fits all’ approach. Thus, perhaps the key to enhancing patient outcomes is through a cross-modal approach that combines the ‘best of both worlds’.

A combined approach of exciting neural tissue with NIBS, followed by behavioural intervention has been advocated. Indeed, some studies have demonstrated that this paired approach produces better outcomes than traditional dysphagia therapy alone [53, 58]. Marchina et al. [53] conducted a systematic review and metanalysis to investigate the effects of tDCS on dysphagia recovery following stroke. Following a comprehensive search of the relevant databases, seven RCT were included in the study analysis. All studies compared anodal stimulation with sham while performing swallowing exercises. Of the total 217 sample size, 115 patients received stimulation and 102 patients sham. Overall, results indicate a small but significantly pooled effect size (0.31; CI 0.03, 0.59; *p* = 0.03), signifying that tDCS with exercises were superior to behavioural rehabilitation alone.

Similarly, Ünlüer et al. [58] compared the effects of a combined low-frequency rTMS/traditional dysphagia exercise protocol to conventional exercises in isolation. These included oropharyngeal muscle strengthening exercises, thermal tactile stimulation, Masako and Mendelson maneuvers, vocal cord exercises, Shaker exercises, and tongue retraction exercises [58]. Each group underwent conventional dysphagia therapy, 3 days per week, for 4 weeks. Additionally, the study group received 1 Hz rTMS to the unaffected hemisphere in the final week [58]. A videofluoroscopic swallowing study (VFSS) was conducted before and after treatment to assess the swallowing functioning of each patient. Following treatment, swallowing function was similar between the groups. However, substantial improvements were seen for appetite, fear of eating, and quality of life measures in the study group compared to the control (*p* < 0.05) [58]. The authors hypothesised that application of rTMS likely produced a positive effect on mood [58]. Of note, the rehabilitation maneuvers in this study were focused on muscle strengthening, with the exception of thermal stimulation. So although active rehabilitation was focused on swallowing, it leads again to the question, is muscle strengthening always the correct approach? Would cortical stimulation maximise peripheral muscle strengthening or would this be more effectively paired with a type of rehabilitation focused on motor control?

Erfmann et al. [59] explored the effects of tDCS on motor skill learning in swallowing for healthy adults. In this study, 39 participants were randomly assigned to either sham, anodal tDCS, or cathodal tDCS conditions. Following 20 min of the midline cerebellar tDCS or sham, participants underwent swallowing skill training using surface electromyography (sEMG) biofeedback to target control of timing and magnitude of submental muscle activation [59]. Since much of the neurophysiological and behavioural studies on cerebellar tDCS have indicated opposing effects for the anodal vs cathodal protocols [60–63], the authors hypothesised that anodal cerebellar tDCS would enhance immediate motor skill learning and effects post training. Conversely, cathodal cerebellar tDCS was predicted to inhibit motor skill learning in swallowing [59]. Despite this, results demonstrated that both anodal and cathodal tDCS had a relative inhibitory effect on motor skill learning in swallowing when compared to the sham condition, which revealed significant improvements in skilled behaviour [59]. Since these data were derived from healthy volunteers, the results must be interpreted with caution. Nonetheless, this research aligns with the findings of Cheng and Hamdy [49] by recognising the discrepancies in the literature related to dysphagic patient responsiveness to NIBS [49].

Although NIBS has demonstrated potential for adapting swallowing, the comprehensive training required to safely and effectively administer NIBS is a deterrent to generalised application and transition to clinical practice [64]. Perhaps then it is wise to turn attention to treatments that are more easily implemented in the clinical environment and beyond. Such treatment options should focus on the principles of neural plasticity to promote intrinsically driven neural change and thereby capitalise on rehabilitation efforts and optimising functional outcomes [42].

## Swallowing Skill Training as a Mechanism for Cortical Change

Skill-based training—as an alternative to muscle strengthening—has been identified in the physical medicine and rehabilitation literature to result in adaptive neural change. Studies have shown motor skill training can result in changes to areas of motor representation within the primary motor cortex, as well as increased synaptogenesis and intracortical connections [65–68]. Additionally, Jensen et al. [69] reported increased motor-evoked potentials measured at the periphery as a function of skill-based training, suggesting more efficient transfer of cortically generated neural information to lower motor neurons. Since swallowing is a motor sequence that is now understood to receive substantive input from the level of the cortex, it is conceivable that such principles of skill training are translatable to swallowing motor control. Cohen et al. [70] proposed that neuroplasticity describes the ability of the brain to change. As discussed, skill training results in such neural change. Thus, the presumption is that skill training, utilised as a rehabilitative approach, will focus on adapting neural input for modification of biomechanical movement.

In identifying and understanding the role of the cortex in swallowing, perspectives may then logically shift to reframe ingestive swallowing as a complex, goal-directed, spatiotemporal task. In this context, how might we define swallowing skill? Swallowing skill for patients and clinicians is ultimately defined as the ability to safely manage secretions and ingest a varied diet without pulmonary compromise, and with enhanced quality of life. To accomplish this functional goal, a more specific and, in the short term, theoretical goal would aid in framing the development of new rehabilitation approaches. Swallowing skill may be defined as the ability to voluntarily modulate the timing, force, and/or coordination of multiple muscles in the performance of this task, resulting in efficient ingestive behaviour which successfully adapts for variations in bolus size, consistency and other variables. As such, swallowing skill training may serve as the direction in which swallowing rehabilitation is indicated for individuals with impaired swallowing biomechanics that are not the result of muscle weakness, rather the result of impaired motor control. In theory, the literature would appear to support the emergence of swallowing skill training approaches, but is there any evidence for cortical modulation of swallowing through behavioural engagement?

## Evidence for Cortical Engagement with the Pharyngeal Response

Remarkable novel research highlights the therapeutic potential of engaging the perceptual and cognitive processes involved in swallowing to rehabilitate dysphagia [71, 72]. For example, the role of action-observation has been discussed to promote functional recovery of swallowing following stroke. AO involves the patient watching a video of a particular action (e.g. swallowing) in order to shape their own response [71]. This process is enabled through mirror neurons, found throughout the brain, that allow for action recognition, understanding, and motor learning [73–75]. Essentially, mirror neurons fire during observation of others performing an action as well as during execution of the task. Therefore, it is believed that this neuronal population may promote plasticity-related functional recovery following brain injury [76, 77].

Jing et al. [71] investigated the role of AO in swallowing using functional magnetic resonance imaging (fMRI). The authors previously identified that some of the brain areas recruited while swallowing overlap with mirror neurons in the motor regions, inferior frontal gyrus (IFG), inferior parietal lobule (IPL) and others [71]. To investigate, 29 healthy participants underwent MRI scanning under three visual conditions: a video of a man biting into an apple, chewing, and swallowing (described as AO), a neutral picture with the word "watching", the same neutral picture with the word "swallowing". When the word "swallowing" was presented on screen, participants were told to execute their natural, comfortable swallowing motion [71]. Two brain regions containing mirror neurons—the left supplementary motor area (SMA) and left middle temporal gyrus—were similarly activated during the AO and execution of swallowing conditions [71]. Therefore, the authors suggest that AO may promote reorganization of cortical networks to improve dysphagia [71]. Although promising, the study results must be considered cautiously since the participants were young and healthy (mean age = 22.76 ± 2.63 years) [71].

Using a very different approach, Szynkiewicz et al. [72] report the effects of a novel, 6-week mental-practice (MP) lingual strengthening protocol in typically ageing adults. This population was intentionally selected since the prevalence of dysphagia increases with age [78]. MP is accomplished through the repetitive rehearsal of a motor task in working memory to facilitate motor improvement [72]. Six healthy participants, aged 53–78 years, were instructed to imagine completing tongue strengthening exercises. Participants began the mental lingual exercises at an imagined 60% peak lingual pressure effort and progressed to an imagined 80% resistance load. Following the MP regime, all participants significantly increased their tongue strength compared to baseline [72] as indicated through mean peak tongue pressure (kPa) using the Iowa Oral Performance Instrument ® (IOPI, Northwest Co., LLC, Carnation, WA, USA IOPI).

To expand on these findings, Szynkiewicz et al. [79] conducted a randomised controlled trial (RCT) to compare physical and mental lingual exercise for healthy older adults. Twenty-nine participants were assigned to one of four intervention groups: physical lingual exercise, physical/MP lingual exercise, MP lingual exercise, or a control group (placebo exercise) [79]. The physical lingual exercise condition required participants to push with peak force against a tongue depressor with resistance (10× reps of each set: protrusion, elevation, left and right lateralisation). The MP protocol had participants imagine themselves completing these exercises, without moving muscles of the tongue, face, head, or neck. The control group completed physical jaw exercises and visualised relaxation exercises to account for the time spent completing the MP component [79]. Each participant completed 3 exercise sessions a day, 3 days per week, for 6 consecutive weeks. Maximum isometric pressure (MIP) and regular effort saliva swallowing pressure were collected at baseline and weeks 2, 4, and 6 [79]. Results indicate that the only group to demonstrate treatment effect from baseline to week 6 was the physical/MP lingual exercise group. Previous findings indicate that strength gains following limited treatment are mainly attributed to central adaptions [80]. This viewpoint supports findings of the current study, since the only intervention to demonstrate significant change in outcomes was the condition that included a cognitive correlate to the physical repetitions. Altogether, these findings highlight the importance of engaging the cortex in swallowing rehabilitation and demonstrate that MP may be a beneficial tool to include in the dysphagia rehabilitative framework.

The Mendelsohn maneuver is commonly recommended in dysphagia management and incorporates some of the neuroplasticity principles by focussing more on the practice of swallowing as a skilled motor performance [14]. This technique aims to alter aspects of pharyngeal phase swallowing by prolonging upper esophageal sphincter (UES) opening [81]. Peck et al. [82] studied the neural network recruited while performing the Mendelsohn maneuver to increase understanding and potentially exploit the therapeutic outcomes associated with this technique [82]. The data indicated increased cortical activation in healthy adults (*n* = 10) when performing the Mendelsohn compared to normal swallowing [82]. Activation was primarily seen in the superior and middle frontal gyrus, angular gyrus, cingulate gyrus, and inferior parietal lobe [82]. Such extensive neural activation as a result of this maneuver is presumably explained by virtue of the technique modulating timing aspects of the swallow. This theory may be especially true since the Mendelsohn maneuver also produced enhanced activation of the SMA[82]. This area has been linked to the initiation of motor control in swallowing [83] and therefore, the increased activity likely correlates with the increased motor involvement of the submental and infrahyoid muscles during the maneuver [82].

The previous studies highlight the powerful role of imagination and cortical thought to activate the swallowing neural network and to induce change in swallowing motor behaviour. Further research identifies increased neural activation during known rehabilitation approaches. Yet to be fully clarified is if increased cortical activation translates to improved swallowing function in dysphagic patients and if rehabilitation approaches can be further developed to maximize cortical modulation of swallowing.

## Volitional Modulation of Swallowing En Bloc

Following from the Peck et al. [82] study, McCullough et al. [31] and McCullough and Kim [84] studied the functional effect of Mendelsohn maneuver to produce lasting changes in swallowing biomechanics. Initially, McCullough et al. [31] recruited 18 outpatients to participate in the crossover study which compared two weeks of treatment with two weeks of no treatment [31]. Each participant was between 6 weeks and 22 months post-stroke. During treatment weeks, individuals were seen twice daily, for one-hour sessions, performing 40 trials of the Mendelsohn maneuver. Participants received visual biofeedback through surface electromyography (sEMG) to guide the target process of maintaining maximum position of the larynx during swallowing [31]. Measures of swallowing duration, penetration/aspiration, residue, and dysphagia severity were analyzed to compare treatment and no-treatment weeks. VFSS data indicated the only significant changes were measures of duration of superior and anterior hyoid movement after 2 weeks of treatment [31]. Although it is plausible that intensive treatment induced changes in central timing measures, it is perhaps more likely that the prolongation of hyoid movement reflects an overall prolongation of the swallowing response, rather than a specific cortical adaptation of hyoid biomechanics.

McCullough and Kim [84] used data from the same crossover design to evaluate the effects of the Mendelsohn maneuver on the extent of hyoid movement and mean width of UES opening. Overall, results indicated the only measure to demonstrate statistically significant gains was hyoid maximum elevation (HME) [84]. This outcome suggests that the intensive treatment may have increased muscle strength in the anterior belly, mylohyoid and geniohyoid muscles, in keeping with the aim of the maneuver. Previous research indicates that strength gains following such limited treatment may be attributed to transient change at the central level to initiate increased muscle activation [80, 85], thus explaining the increased activation identified in the MRI study by Peck and colleagues [82]. However, data from McCullough and Kim [84] do not strongly suggest a significant and long-term alteration of the swallowing motor plan; rather it suggests a key rehabilitation outcome of peripheral muscle activation.

Guedes et al. [86] examined transfer effects of the volitional laryngeal vestibule closure (vLVC) maneuver to natural swallowing in healthy participants. The vLVC largely aligns with the Mendelsohn maneuver, as it begins with a swallow and requires the individual to extend laryngeal vestibule closure (LVC) for a minimum of 2 s [86]. Two discrete studies were conducted to investigate the transference of different tasks. The long-hold vLVC required participants to prolong swallowing for as long as possible, whereas in short-hold vLVC, participants were asked to perform 2-s vLVC swallowing [86]. For long-hold vLVC training, participants completed 7 trials, whereas in the short-hold training condition, 20 vLVC swallows were executed. All swallows were recorded with VFSS. Detailed kinematic analysis revealed faster laryngeal vestibule closure reaction time (LVTrt) transferred to post-training 5 ml liquid swallows, for both training conditions [86]. These findings are clinically significant since delayed LVC poses a significant risk of aspiration [87]. As there was no evidence of increased total LVC duration which is what it was designed to achieve [86], rather a change in timing of reaction time, this may suggest some adaptation in the overall swallowing motor plan.

Focussing on a different physiologic feature of swallowing, Nativ-Zeltzer et al. [88] evaluated patients’ ability to volitionally control the UES through use of high-resolution manometry (HRM) biofeedback. The study patients (*n* = 10) included were undergoing HRM for evaluation of dysphagia, globus, chronic cough, and gastroesophageal reflux [88]. The patients practiced adjusting the pressure at the UES in response to a color-coding system (e.g., warmer colors represented UES elevation, cooler colors represented UES relaxation). During a single training session, participants were instructed to sustain 30 s of UES tightening and 30 s of UES relaxation, with 1-min resting periods between the tasks. Outcomes were mixed. Participants were able to increase UES pressure with biofeedback for both the mean (30.1 (± 15.3) mmHg to 44.8 (± 25.03) mmHg (*p* = 0.02)) and maximum (63.84 (24.1) mmHg to 152.4 (123.7) (*p* = 0.04)) [88]. Although some participants were able to decrease basal UES tone, no statistically significant effect was seen across the group (*p* > 0.05) [88] for this task.

These preliminary findings suggest that it is possible to train volitional control of UES pressure as a single task, with HRM driven biofeedback, albeit only by increasing pressure. Given that the training protocol was restricted to a single session, the findings likely indicate short term behavioural adaption rather than change at the level of the brain. Additional research was indicated to identify if intensive training could facilitate neural change to control UES pressure during the act of swallowing. Of particular interest was the ability of participants to decrease UES pressure, since dysphagic patients more often present with failure to relax the UES [89]. Collectively, these studies emphasize the influence of cortical control to modulate pharyngeal swallowing ‘en bloc’.

Guedes et al. [86] focused on volitional laryngeal vestibule closure and noted that it possible to alter this component of the pharyngeal response. Yet, this modification was not considered in isolation. Nativ-Zeltzer et al. [88] found that patients were able to volitionally control UES pressure. However, since this was not investigated during swallowing, the effects of this behavioral adaption on the pharyngeal response are unknown. Therefore, according to these previous findings, it is not possible to confirm whether individual aspects of the swallowing response can be deliberately modulated. For example, coaching participants to swallow longer may have merely altered the length of the motor response rather than laryngeal vestibule closure in isolation. The important question that remains pertains to whether individuals are truly able to adapt the swallowing motor plan and thereby control and change isolated components of the pharyngeal swallow.

## Volitional Modulation of Isolated Components of the Swallowing Motor Plan

When considering this issue, it is important to reflect on the motor speech literature and the concepts of knowledge of performance (KP) and knowledge of results (KR) [90]. This type of feedback indicates to a patient how an action was performed and their success in executing the task [90]. Both theoretical underpinnings are helpful when relearning a motor response [90]. An obvious inherent challenge associated with learning to adapt pharyngeal swallowing is the difficulty in “seeing” this behaviour. Alike some of the previous studies, the following research has addressed this limitation by integrating biofeedback to guide participants’ learning in order to modulate swallowing.

Elaborating on the focus of Nativ-Zeltzer et al. [88], Winiker et al. [91] explored the potential for behavioural UES pressure adaption during swallowing in healthy adults. Participants in this study were asked to increase the period of pressure drop in the region of the UES (UES-Pdrop) without altering swallowing biomechanics [91]. Six healthy females aged between 23 and 68 years (mean age of 36 years) attended daily, 45-min sessions, for 2 weeks (10 days) [91]. Participants were initially familiarized with major landmarks and points of interest on the high-resolution manometry (HRM) contour plots. Subsequently, they were asked to self-explore methods to prolong the duration of UES opening through pressure manipulation at the UES itself, and avoid altering the overall swallowing pattern [91]. In total, 64 saliva swallows were performed each session, with regular breaks between. Baseline and outcome measures were recorded during performance of natural swallows (e.g., five saliva and five cup-sip water) followed by manipulated swallows, whereby the participants were asked to increase the duration of pressure drop [91]. Initial assessment revealed no overall change in pharyngeal dynamics as measured by pharyngeal timing and pressure. Evaluation of UES pressure revealed that participants were able to volitionally prolong the period of UES pressure drop within the initial training session. However, interestingly, additional training did not further enhance performance [91]. The authors proposed that this may have been due to the innate capacity of individuals to increase UES opening duration, and therefore extend the period of UES-P drop [91]. This finding highlights the potential limits of cortical control to override the swallowing motor plan, which is responsible for opening the UES [37, 91]. This, however, may be true only in healthy participants with normal swallowing, thereby limited in their ability to modulate this already functional response [91].

Despite these limitations, this study demonstrates that it is possible to provide limited modulation of pressure at the UES without altering additional pressure patterns of pharyngeal swallowing [91]. To further understand the true therapeutic potential of this training, it should be applied to patients with dysphagia who have greater scope to modulate their impaired swallowing [91].

Lamvik et al. [92] investigated the capacity of healthy participants to modulate the latency of pharyngeal closure in isolation. The fundamental aim of this exploratory study was to determine if healthy humans could learn to alter the ‘reflexive’ component of the swallowing response [92]. All healthy participants (*n* = 6), aged between 19 and 44 years (mean = 29 years), underwent a total of 10 one-hour sessions, across a two-week treatment period [92]. Participants were coached on the training objective that was to reduce the separation between the peaks of upper and lower pharyngeal sensors. This was visualized through the use of pharyngeal manometry, linked to a computer monitor presenting manometric waveforms as biofeedback. A blue line on the monitor represented pressure generation in the proximal pharynx and a red line represented pressure in the distal pharynx [92]. Each session started with collection of pre-training baseline swallows, to monitor the training effect on participant’s swallowing motor plan. This was followed by three 15-min blocks of training and then by post-training swallows, executed without biofeedback [92]. As these were normal swallowing participants, during the training blocks, they were instructed to adapt their swallowing behavior to produce an abnormal, mis-sequenced response—by making the red line come before the blue, or by making the waveforms overlap. In the post-training component, participants were asked to produce five of their best mis-sequenced swallows [92]. The results found that participants were able to significantly reduce the temporal separation between the waveforms, including during the post-training protocol exclusive of visual feedback. However, no further reductions were achieved during the second week of the program [92]. This finding suggests that there may be a protective limit in the ability of healthy participants to intentionally maladapt pressure generation in the pharynx while swallowing. Another important outcome was that change in pharyngeal pressure latency was moderately corelated with change in swallowing duration and amplitude. Notably, post training swallows revealed a reduction in swallowing duration [92]. By in large, this suggests that participants were likely modulating total swallow duration to achieve the goal rather than altering the latency of pharyngeal functioning in isolation. The authors indicate that future research should investigate the training effect as it relates to dysphagic patients’ ability to control targeted aspects of the pharyngeal swallow [92].

Huckabee et al. [13] recruited patients with atypical dysphagia to participate in intensive rehabilitation with HRM driven biofeedback. The study participants (*n* = 16) presented with a phenomenon described as pharyngeal mis-sequencing [13], characterized by impaired timing of pressure generation at the proximal and distal pharyngeal region, potentially resulting in nasal redirection, aspiration, and inability to tolerate a normal diet [13]. As per the study protocol, all patients participated in 1-h treatment sessions, twice daily, for a minimum of 1-week. Ongoing intervention was provided for an additional week according to patient availability [13]. During treatment, participants were instructed to swallow and while doing so, volitionally increase the temporal separation between the upper and lower pharyngeal pressure waveforms. This was visualized using pharyngeal manometry and indicated by the blue line (proximal sensor) coming before the red line (distal sensor) as much as possible [13]. Following treatment, the average latency between peak pressures at the proximal and distal pharynx increased from a pre-treatment mean of 15 ms (95% CI − 2 to 33 ms) to a post-treatment mean of 137 ms (95% CI 86–187 ms). This change in pressure was associated with 11/16 patients returning to a normal diet, a substantial outcome given that 6 patients presented with chronic dysphagia prior to treatment and relied on percutaneous endoscopic gastrostomy (PEG) for total nutrition [13]. Of note, of the 5 patients who did not return to full oral diet, 4 were unable to continue rehabilitation beyond one week, suggesting the need for intensity and repetitions in treatment. The findings translate to improved clinical care and indicate that biofeedback can be used to modify specific and isolated components of the pharyngeal response and thereby, improve functional swallowing outcomes in patients with dysphagia [13].

Martin-Harris et al. [93] sought to improve swallowing impairments in the head and neck cancer (HNC) population by training safe and efficient respiratory-swallowing patterns [93]. Previous research led to the current work by indicating that unstable respiratory-swallowing coordination in this population poses risk to airway invasion and swallowing disorders [94]. The training protocol was sectioned into 3 learning modules: identification of respiratory-swallowing patterns, acquisition using biofeedback to produce the optimal respiratory pattern during liquid swallows, and mastery, to ensure the appropriate expiratory-swallow-expiratory pattern was achieved without visual or verbal feedback to 80% accuracy. Respiratory-swallowing coordination was trained using simple graphic illustrations displayed on the KayPENTAX Digital Swallowing Workstation [93]. HNC patients attended twice weekly, 1 h training sessions, to achieve mastery of the optimal respiratory-swallowing pattern. All participants were able to complete the intervention within 4 weeks (range, 4–8 sessions). On conclusion of the protocol, all patients were able to learn and implement the optimal respiratory-swallowing pattern following treatment (*p* < 0.001). These gains in motor skill correlated with improvements in VFSS measures, including laryngeal vestibule closure (LVC) (*p* < 0.004), tongue-base retraction (*p* < 0.001) and a reduction in pharyngeal residual (*p* = 0.01). Likewise, significant improvements were noted in Penetration-aspiration scale (PAS) scores (*p* < 0.0001). Moreover, the improvements were maintained in participants who attended the 1-month follow-up [93]. While some of the treatment outcomes are intuitive (e.g. LVC; improved PAS), changes in tongue-base retraction and pharyngeal residual are intriguing since these components were not directly trained. Kleim and Jones [42] describe such events through the principle of transference which refers to the ability of “plasticity within one set of neural circuits to promote concurrent or subsequent plasticity” (p. 8).

Taken together, these results contribute to the increasing evidence that it is possible to modify individual components of the pharyngeal swallow, particularly in patients with dysphagia. The studies also highlight the crucial role of instrumentation to provide patients with biofeedback, upholding the principles of KP and KR [90]. Although this procedure has many merits, it is an invasive option. Especially when it is considered for intensive or long-term rehabilitation [95]. The approach of Martin-Harris and colleagues [93] provided patients with biofeedback through less invasive methods, with evidence of transference in treatment effects to other swallowing behaviour.

Athukorala et al. [96] evaluated a less specific skill-training approach using sEMG to detect timing and magnitude of submental muscles when swallowing. Ten patients with dysphagia secondary to Parkinson’s disease completed two weeks of one-hour daily treatment [96]. The objective was to improve the precision of swallowing muscle contraction by developing conscious control over the timing and relative strength of swallowing [96]. Participants were required to “hit” a randomly placed swallowing target, shown when the peak of the time-by-amplitude waveform reached a box on the computer screen. To mitigate the effects of effortful type swallowing, all targets were calibrated between 20 and 80% of maximal submental sEMG amplitude [96]. Task challenge was implemented following three successive ‘hits’ which resulted in a 10% decrease of the target size. Conversely, an automated increase of 10% in target size followed three successive misses. One hundred repetitions of the swallowing task were performed in blocks of ten, with scheduled breaks between. Outcome measures included the Timed Water Swallowing Test (TWST) [97], the Test of Masticating and Swallowing Solids (ToMaSS) [96, 98], and sEMG timing measures of pre-motor (reaction time), pre-swallow (anticipatory movement) and total swallowing duration times. In addition, participants completed the self-reported Swallowing Quality of Life (SwalQOL) survey [99]. Before training, study patients displayed stable performance across a 2-week baseline period. Immediately post-treatment, they demonstrated significant improvement in all measures of the TWST, and sEMG measures during dry and liquid swallows. Although the treatment was based on control of dry swallows, these outcomes suggest transference to liquid boluses. No changes were apparent on measures from the ToMaSS, likely due to the participants' absence of impairment in solid bolus swallowing [96, 98]. Reassessment at 2 weeks post-treatment revealed maintenance [96] of gains following treatment. Despite the small sample size, the functional improvements seen in participants' swallowing provides preliminary evidence of the effectiveness of this novel intervention. Paired with the results of Martin-Harris et al. [93], this study indicates the value of task specific dysphagia intervention (e.g. focused on swallowing) to improve swallowing outcomes. Importantly, these data also support the concept of task transference following skill-based swallowing training.

## Conclusion

Looking back often helps us look ahead by contextualizing where we have been and providing direction for where we need to go. The use of exercises that promote strengthening of the muscles involved in swallowing have long prevailed in the clinical toolbox. With an increasing appreciation of cortical control mechanisms that either modulate (or perhaps directly plan) ingestive behaviour, a pathway is paved to greater options for rehabilitation. Evidence is emerging of the capacity to volitionally enhance or override the swallowing response. This evidence suggests potential for exploiting skill-learning during swallowing.

Skill-based swallowing training has recently emerged as a potential alternate approach to rehabilitation of dysphagia. It is predicated on an assumption of impaired motor planning and execution. But despite early promising results for the capacity to alter individual components of the pharyngeal response through this approach, considerable definitional work is left to be done. If muscle strengthening addresses weakness or flaccidity, what specifically does skill training address? Pathophysiologic neuromotor conditions of apraxia, ataxia and the like might presumably respond to skill-based training. But do these conditions exist relative to dysphagia? If so, how do we define them?

As we move into the next decade of dysphagia management practices, we have an opportunity to further refine our capacity to diagnose and rehabilitate dysphagia. Clinically, we need to move beyond a description of biomechanics—what moves where and when. We now need to focus understanding pathophysiology—what underlying neuromotor impairment explains biomechanical imprecision. In this way, we will ultimately be able to provide more precise, and effective, approaches for rehabilitation of dysphagia.
